# Evaluation of synovium-derived mesenchymal stem cells and 3D printed nanocomposite scaffolds for tissue engineering

**DOI:** 10.1088/1468-6996/16/4/045001

**Published:** 2015-07-16

**Authors:** Jian-Feng Pan, Shuo Li, Chang-An Guo, Du-Liang Xu, Feng Zhang, Zuo-Qin Yan, Xiu-Mei Mo

**Affiliations:** 1 Department of Orthopedics, Zhongshan Hospital of Fudan University, 180 Fenglin Road, Shanghai 200032, People’s Republic of China; 2 State Key Laboratory for Modification of Chemical Fibers and Polymer Materials, College of Materials Science and Engineering, Donghua University, 2999 North Renmin Road, Shanghai 201620, People’s Republic of China; 3 Biomaterials and Tissue Engineering Lab, College of Chemistry, Chemical Engineering and Biotechnology, Donghua University, 2999 North Renmin Road, Shanghai 201620, People’s Republic of China

**Keywords:** synovium-derived mesenchymal stem cells, nanoparticles, 3D printed nanocomposite scaffolds

## Abstract

Stem cells and scaffolds play a very important role in tissue engineering. Here, we isolated synovium-derived mesenchymal stem cells (SMSCs) from synovial membrane tissue and characterized stem-cell properties. Gelatin nanoparticles (NP) were prepared using a two-step desolvation method and then pre-mixed into different host matrix (silk fibroin (SF), gelatin (Gel), or SF–Gel mixture) to generate various 3D printed nanocomposite scaffolds (NP/SF, NP/SF–Gel, NP/Gel-1, and NP/Gel-2). The microstructure was examined by scanning electron microscopy. Biocompatibility assessment was performed through CCK-8 assay by coculturing with SMSCs at 1, 3, 7 and 14 days. According to the results, SMSCs are similar to other MSCs in their surface epitope expression, which are negative for CD45 and positive for CD44, CD90, and CD105. After incubation in lineage-specific medium, SMSCs could differentiate into chondrocytes, osteocytes and adipocytes. 3D printed nanocomposite scaffolds exhibited a good biocompatibility in the process of coculturing with SMSCs and had no negative effect on cell behavior. The study provides a strategy to obtain SMSCs and fabricate 3D printed nanocomposite scaffolds, the combination of which could be used for practical applications in tissue engineering.

## 1. Introduction

Articular cartilage performs a highly specialized cushion function, covering and protecting bone from weight-bearing forces several times the weight of the body. Cartilage defects are increasing due to greater athletic involvement and population aging. Due to lack of access to the blood supply, cartilage defects undergo limited spontaneous repair and progressively deteriorate into osteoarthritis. Thus early treatment of cartilage defects is critical to prevent cartilage degeneration. The typical protocols [1] are autologous chondrocyte transplantation (ACT), microfracture and allogenic osteochondral grafting to ease the discomfort of these injuries and restore mobility. However, these approaches do not regenerate tissue that resembles its native form to restore a normal articular surface. Each biopsy of ACT causes an additional trauma to donor site cartilage and may result in long post-operative recovery time and increased pain [2]. Microfracture has not proven to be as successful as originally predicted [3]. Allogenic osteochondral grafting includes delayed vascular penetration, slow bone formation, high incidence of incomplete integration and possibility of disease transmission [4]. Tissue engineering uses stem cells combined with appropriate biomaterials to aid tissue formation or regeneration, and thereby represents a promising way to solve the aforementioned problems.

Several stem cells have been investigated including induced pluripotent stem cells (iPS cells) [5], embryonic stem cells (ESCs) [6] and mesenchymal stem cells (MSCs) [7]. MSCs, isolated from bone marrow, adipose, periosteum, muscle, and synovium, have been regarded as a promising cell type for the regeneration of these damaged tissues because of their self-renewal and multilineage differentiation capacity. Since synovium-derived MSCs (SMSCs) were identified as a new member of the MSC family in 2001, increasing attention has been placed on these cells [8]. SMSCs maintain a greater proliferative ability and chondrogenic potential than other sources of MSCs. A 100-fold expansion was achieved in 14 days by plating SMSCs at 50 cells per cm^2^ and thus a sufficient number of cells can be obtained from a minimal amount of synovial membrane tissue for clinical use [9]. Their high proliferation rate does not decrease in patients of advanced age. Compared to bone marrow MSCs and adipose MSCs, SMSCs can easily retain multi-potency capacity without losing their differentiation potential after expansion *in vitro* over four or more passages [10]. In our previous study, we fabricated modified dextran–gelatin hydrogels with SMSCs to form cartilage tissue [11, 12].

The value of biomaterials in tissue engineering is their capacity for rapid defect filling and provision of an active environment which could allow local release of biomolecules to facilitate tissue repair. Such scaffolds can be designed and fabricated using additive manufacturing (AM) [13–15] approaches, such as 3D printing [16], rapid prototyping [17] or solid freeform fabrication [18]. Compared with traditional technologies, AM approaches allow complex shapes for scaffold fabrication according to the computer aided design (CAD) image data of patients. It begins with a CAD model generated from a computerized tomography (CT) scanned image of the object, which is then digitized and sliced into model layers with special software. Then the AM system prints 2D layers into a 3D scaffold, adding each new layer on top of the prior layer. In particular, nanoparticles can be pre-mixed into the host matrix to print the framework, because nanoparticles can serve as a kind of drug carrier to improve release durability of the encapsulated drug. If necessary, two or more different kinds of biomaterials can be printed into the framework to mimic various components of native tissues. This availability of control offers a powerful tool to produce 3D printed nanocomposite scaffolds that can frequently be used directly from the printer.

In the present study, we isolated SMSCs from synovial membrane tissue and characterized stem-cell properties such as surface phenotypes, adipogenic, osteogenic and chondrogenic differentiation. Then we prepared gelatin nanoparticles (NP) using a two-step desolvation method and selected silk fibroin (SF) and gelatin (Gel) as the printing media. 3D printed nanocomposite scaffolds (NP/SF, NP/SF–Gel, NP/Gel-1, NP/Gel-2) were fabricated through the marriage of AM and nanotechnology. The biocompatibility of nanocomposite scaffolds was further assessed by coculturing with SMSCs. This work provided a basis for further studies or practical applications of these novel 3D printed nanocomposite scaffolds combined with SMSCs in tissue engineering.

## 2. Materials and methods

### 2.1. Materials

Gelatin (type B) was purchased from Sigma-Aldrich (St. Louis, MO, USA). Cocoons of *Bombyx mori* silkworm were purchased from Jiaxing Silk Co. (People’s Republic of China). Glutaraldehyde (GA, 25 wt% solution in water) was obtained from Sinopharm Chemical Reagent Co., Ltd Fetal bovine serum (FBS), phosphate buffered saline (PBS), Dulbecco’s modified Eagle’s medium (DMEM), collagenase II, penicillin-streptomycin solution and all other culture media and reagents were purchased from Gibco Life Technologies Corporation (Carlsbad, CA, USA). CCK-8 was purchased from Dojindo Corporation (Kumamoto, Japan). Tissue culture plates and flasks were obtained from BD Biosciences Corporation (San Jose, CA, USA).

### 2.2. Culture of SMSCs and characterization of stem-cell properties

#### 2.2.1. SMSCs culture

Synovial membrane tissue was harvested aseptically from the knee joint of New Zealand white rabbits in accordance with the guidelines approved by the animal committee of Fudan University, People’s Republic of China. The synovial tissue collected was rinsed with PBS solution containing antibiotics (100 units ml^−1^ penicillin, 100 units ml^−1^ streptomycin), minced and digested with trypsin-EDTA (a mixture of trypsin and ethylenediaminetetraacetic acid (EDTA): 0.1% trypsin, 0.4 mM EDTA) at 37 °C for 30 min, then digested with 0.1% collagenase II in complete medium (low-glucose DMEM containing 10% FBS and antibiotics) at 37 °C for 2 h. After incubation cells were collected from digested solution with filter, centrifugated at 1500 rpm for 5 min to obtain a cell pellet and resuspended in complete medium at suitable concentration. Then cells were seeded in culture flasks and allowed to proliferate in the complete medium at 37 °C in a humidified atmosphere of 5% CO_2_. The complete medium was replaced once every 3–4 days. When the attached cells reached 90% confluence, they were washed with PBS solution, collected by treatment with trypsin-EDTA and seeded in new culture flasks for the subculture.

#### 2.2.2. Characterization of SMSCs surface phenotypes

Cell surface phenotypes were determined using flow cytometry. Briefly, 1 × 10^6^ SMSCs were incubated on ice in PBS. Then mouse monoclonal antibodies (phycoerythrin-conjugated or fluorescein-isothiocyanate-conjugated) were applied for 30 min, including CD44 (Abcam, Cambridge, MA, USA), CD45 (Abcam, Cambridge, MA, USA), CD90 (BD Pharmingen, San Jose, CA, USA), CD105 (GeneTex, Irvine, CA, USA) and isotype-matched immunoglobulins (IgGs). After washing with cold PBS, the cells were analyzed on a dual laser BD FACSCalibur (BD Biosciences).

#### 2.2.3. Chondrogenic differentiation of SMSCs

SMSCs were cultured in chondrogenic medium consisting of high-glucose DMEM, 40 *μ*g ml^−1^ proline, 100 nM dexamethasone, 100 units ml^−1^ penicillin, 100 units ml^−1^ streptomycin, 0.1 mM ascorbic acid-2-phosphate and 1 × ITS Premix (BD Biosciences) with the supplementation of 10 ng ml^−1^ TGF-*β*3 for 28 days. Chondrogenic differentiation was assessed using immunohistochemistry stain for collagen II protein.

#### 2.2.4. Adipogenic differentiation of SMSCs

SMSCs were cultured in adipogenic medium consisting of growth medium (low-glucose DMEM, 10% FBS, 100 units ml^−1^ penicillin, 100 units ml^−1^ streptomycin) supplemented with 10^−6^ M dexamethasone, 0.5 mM isobutyl-1-methy xanthine, 100 *μ*M indomethacin and 10 *μ*g ml^−1^ insulin for an additional 21 days. Adipogenic differentiation was assessed using Oil Red O stain for adipose oil.

#### 2.2.5. Osteogenic differentiation of SMSCs

SMSCs were cultured in osteogenic medium consisting of growth medium supplemented with 10^−7^ M dexamethasone, 10 mM *β*-glycerol phosphate, 0.2 mM L-ascorbic acid and L-glutamine for an additional 21 days. Osteogenic differentiation was assessed using alizarin red stain for extracellular matrix (ECM) calcification.

### 2.3. Preparation and characterization of 3D scaffolds

#### 2.3.1. Preparation of nanoparticles

Gelatin nanoparticles (NP) were prepared using a two-step desolvation method as reported previously [19]. First, 5 g of gelatin was dissolved in 100 ml deionization water at 50 °C. Then 100 ml of acetone was added to gelatin solution and stirred for 2 min After 1 min the supernatant was discarded and the sediment containing high molecular weight gelatin was redissolved in 100 ml deionization water at 50 °C. The pH of gelatin solution was adjusted between 2 and 3 with HCl. Under constant stirring at 50 °C, 300 ml of acetone was added dropwise (less than 3 ml min^−1^) into the solution to form gelatin nanoparticles. After the addition of acetone, the suspension was cross-linked with glutaraldehyde (GA, 25 wt% solution in water) overnight at room temperature. Then the organic solvent of nanoparticles suspension was evaporated by a rotary evaporator. The pH of nanoparticles in water was adjusted to 7. Finally, they were subjected to centrifugation (12 000 g for 5 min) and freeze-dried for 24 h. The morphology of nanoparticles was visualized by using scanning electron microscope (SEM, JSM-5600, Japan).

#### 2.3.2. Fabrication of 3D scaffolds

Raw silk was degummed three times with 0.5 wt% Na_2_CO_3_ solution at 100 °C for 30 min each time and then washed with distilled water. Degummed silk was dissolved in a ternary solvent system of CaCl_2_/H_2_O/EtOH solution (molar ratio 1/8/2) for 1 h at 70 °C. The solution was dialyzed with cellulose tubular membrane (250-7u; Sigma) in distilled water for 3 d at room temperature. The water was exchanged every 4 h. The SF solution was filtered and lyophilized to obtain the regenerated SF sponges. After that SF solution (SF, 20%, w/v), SF and gelatin mixed solution (SF–Gel, 10% SF plus 10% Gel, w/v) and gelatin solution (Gel, 10% and 20%, w/v) were prepared as host materials matrix. Then, 3 g of gelatin nanoparticles was dispersed in 10 ml of host solution under stirring to form blended gels at 40 °C. The composition of 3D nanocomposite scaffolds is shown in table 1. A MAM (Motor Assisted Microsyringe, Fochif Mechatronics Technology Co., Ltd) system was employed to construct 3D porous scaffolds. Blended gels were loaded into the extrusion chamber and heated to 40 °C. Then they were deposited on a platform through a 21G nozzle in a layer-by-layer fashion. The lay-down pattern of the scaffolds was set at 45/−45° and square pores were created. The distance between center lines of two adjacent parallel strands was set at 1 mm.

**Table 1.** Composition of 3D nanocomposite scaffolds.

Scaffolds	Nanoparticles (NP) (w/v), %	Silk fibroin (SF) (w/v), %	Gelatin (Gel) (w/v), %
NP/SF	30	20	
NP/SF–Gel	30	10	10
NP/Gel-1	30	0	20
NP/Gel-2	30	0	10

#### 2.3.3. Crosslinking of scaffolds

After fabrication NP/SF and NP/SF–Gel scaffolds were treated with 75% ethanol vapor for 6 h to convert the SF conformation from a random coil or *α*-helix to a *β*-sheet structure. Then NP/SF–Gel, NP/Gel-1, and NP/Gel-2 scaffolds were firstly crosslinked in the GA vapor for 48 h at room temperature. After that they were transferred into the GA solution and crosslinked again at 4 °C for 48 h. The crosslinking process was carried out between the amine groups of gelatin and aldehyde groups of GA. The residual aldehyde groups of the scaffolds were removed by soaking into 100 mM glycine solution. Then all scaffolds were dried in a vacuum at room temperature for 24 h.

#### 2.3.4. SEM observations

To characterize the microstructures of 3D scaffolds, samples were cut to expose the cross-sections and coated with gold for 120 s using a sputter coater. The samples were observed by using a scanning electron microscope (SEM, JSM-5600, Japan) under an accelerating voltage of 10 kV.

### 2.4. Stem cells assessment cocultured with 3D scaffolds

The cell viability and proliferation was assessed using CCK-8 assay at 1, 3, 7 and 14 days through cocultured with 3D scaffolds in Transwell system (Costar 3422). Briefly, SMSCs suspension with density of 1 × 10^6^ cells ml^−1^ was injected in 24-well culture plates. 3D scaffolds were placed within the upper chambers. Then the upper chambers were inserted in 24-well culture plates and the complete medium was added. SMSCs seeded in a 24-well culture plate without upper chamber served as control group. The culture plates were incubated at 37 °C in a humidified atmosphere of 5% CO_2_. The culture medium was exchanged every three days. After 1, 3, 7 and 14 days culture, the culture medium was removed and 400 *μ*l medium containing 40 *μ*l CCK-8 reaction solution was added to each well and incubated for 4 h at 37 °C. Then the medium with CCK-8 was transferred to 96-well tissue culture plate and the absorbance was read at 450 nm using a multidetection microplate reader (MK3, Thermo, USA). All experiments were carried out in triplicate. Then SMSCs were cocultured with 3D scaffolds for 14 days in a tissue culture plate and observed under a phase contrast microscope for cell behavior.

### 2.5. Statistical analysis

Tests were done in three replicates, unless otherwise stated. All quantitative data were recorded and statistically analyzed by SPSS 19.0. Values were expressed as the mean of three replicates and standard deviation (SD). Experimental results were also analyzed by one-way ANOVA. For all statistical tests, the level of significance was set at *p* < 0.05.

## 3. Results

### 3.1. SMSCs surface phenotypes

**Figure 1.**
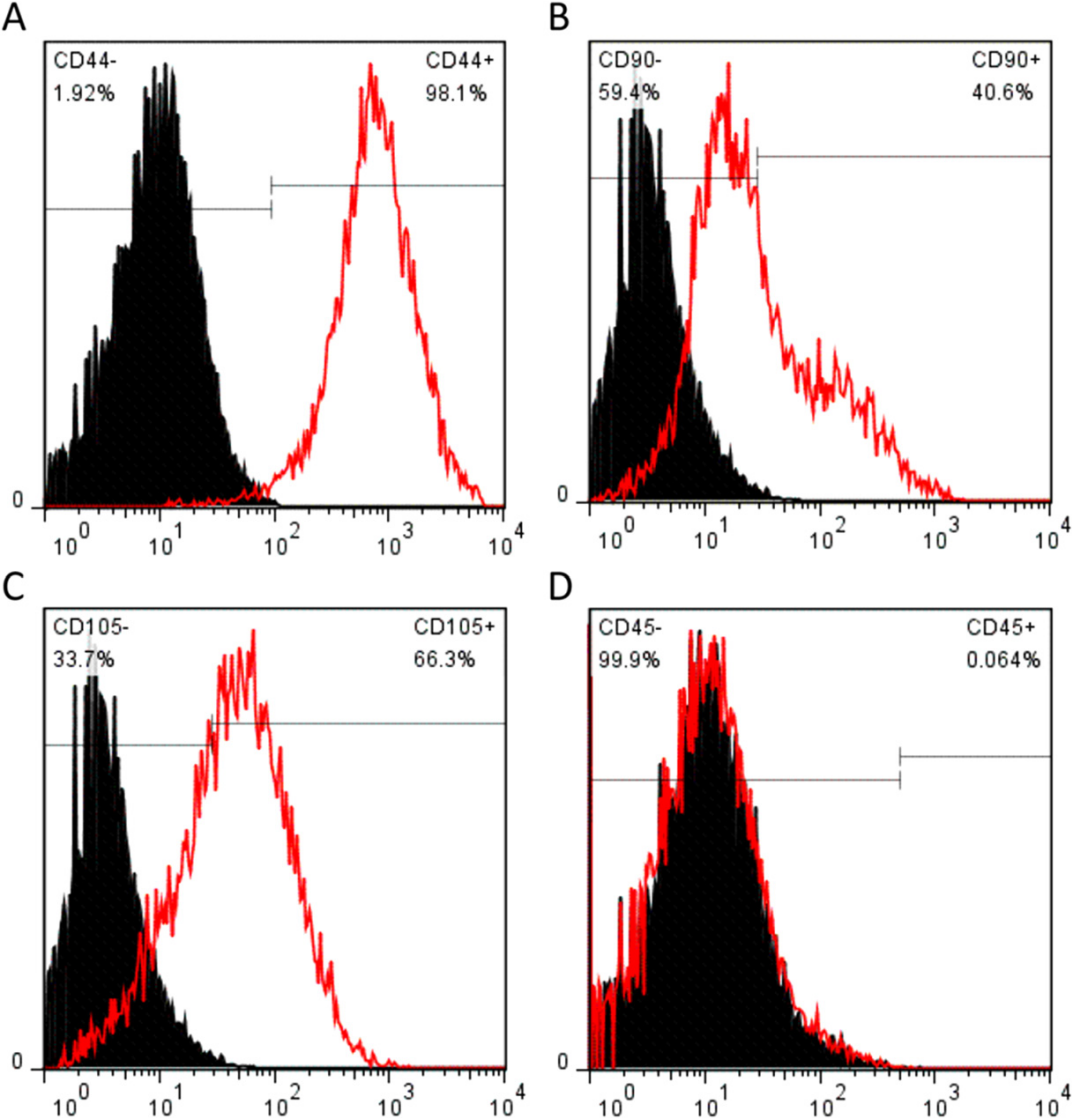
Quantitative flow cytometry analysis of typical cell surface markers (red color) from mesenchymal stem cell (CD44, CD90, and CD105) (a)–(c) and hematopoietic stem cell (CD45) (d).

The SMSCs were characterized for the phenotype expression profiles including MSC surface markers (CD44, CD90, and CD105, figures 1(a)–(c)) and a hematopoietic stem cell (HSC) marker (CD45, figure 1(d)). MSC surface markers CD44, CD90, and CD105 showed positive expression in SMSCs. In contrast, there was no HSC marker CD45 detectable in the SMSCs. The percentage of CD44-positive SMSCs was 98.1%. An important indicator of chondrogenic potential, the CD90 data (40.6%) suggested that SMSCs have the capacity for differentiating toward chondrogenesis. These positive markers, CD44, CD90, and CD105, are not unique to SMSCs. Being a new member of the MSC family, SMSCs are similar in their surface epitope expression to other MSCs. Bone marrow–derived MSCs (BMSCs) are negative for CD45 and positive for CD44, CD90 and CD105, which is similar for SMSCs in this study. There is little difference in surface epitopes between SMSCs and BMSCs, and to date no specific marker has been found to positively identify SMSCs.

### 3.2. SMSCs multilineage differentiation

**Figure 2.**
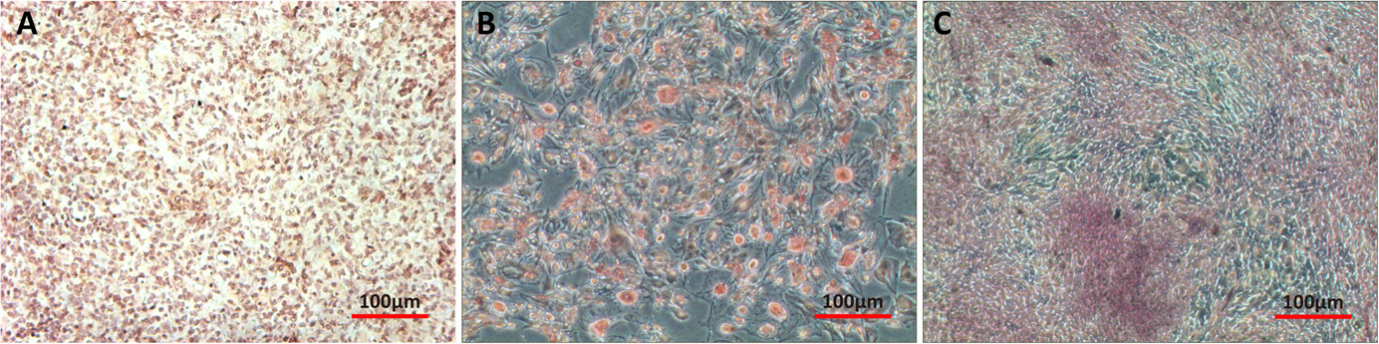
Multilineage differentiation of SMSCs. (a) After incubation in chondrogenic medium SMSCs were positively stained for collagen II protein (immunostaining). (b) After incubation in adipogenic medium SMSCs were positively stained for adipose drops (Oil Red O). (c) After incubation in osteogenic medium SMSCs were positively stained for calcium matrix (Alizarin Red).

Chondrogenic differentiation of SMSCs was demonstrated by the accumulation of collagen II. Positive staining for collagen II protein immunohistochemistry confirmed the cartilage phenotype of SMSCs after differentiation (figure 2(a)). SMSCs incubated with adipogenic medium underwent a change in their morphology from spindle-shaped to intumescent and formed large adipose drops that were positively stained by Oil Red O (figure 2(b)). Osteogenic medium–treated SMSCs displayed bone-specific metachromasia with positive Alizarin Red staining, which is specific for the calcium matrix, a unique biochemical feature of bone nodules (figure 2(c)).

### 3.3. Characteristics of nanoparticles

**Figure 3.**
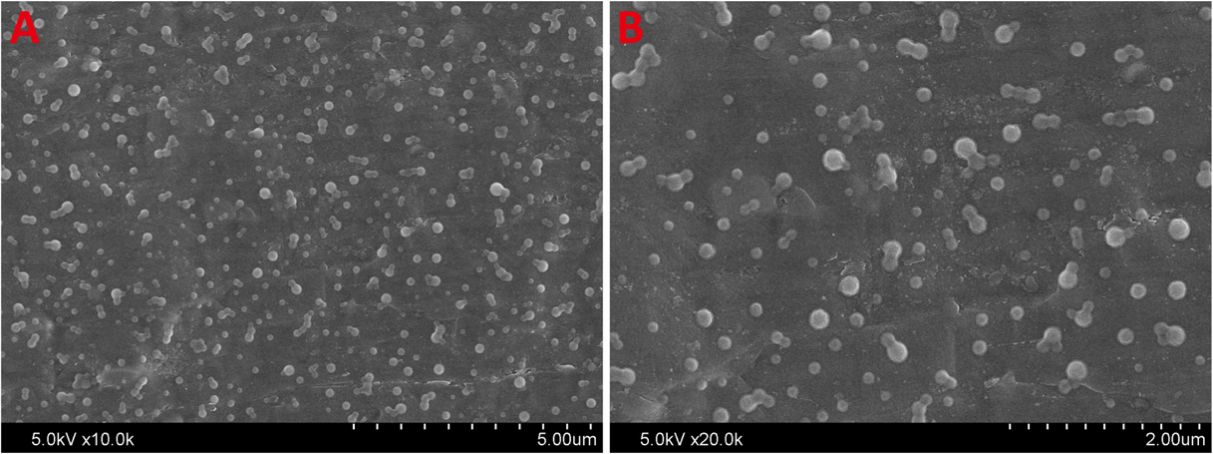
SEM images of gelatin nanoparticles.

Scanning electron microscope (SEM) was utilized to visualize the morphology of lyophilized gelatin nanoparticles (figure 3). The average diameter of nanoparticles was 320 ± 18 nm and the polydispersity index was 0.33 ± 0.04. Nanoparticles or nanospheres have been considered as ideal containers and barriers of drug molecules due to their high surface-area-to-volume ratio, affording the drug with a sustained release profile. Superior characteristics of gelatin include beneficial biological properties comparable to collagen, ease of processing into microspheres, gentle gelling behavior, controllable degradation characteristics by tailoring crosslinking conditions, and abundant presence of functional groups that allow for further functionalization and modification via chemical derivatization. These properties make gelatin optimal for use as a delivery vehicle for drugs or proteins. Therefore, it is quite reasonable to design gelatin nanoparticles possessing high surface-area-to-volume ratio, high surface activity, good biocompatibility, and strong ability to absorb a variety of drug molecules.

### 3.4. Characterization of 3D scaffolds

**Figure 4.**
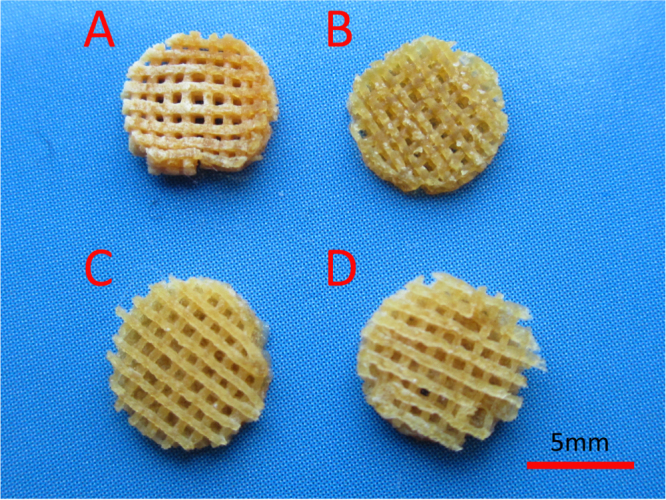
3D printed nanocomposite scaffolds. (a) NP/SF, (b) NP/SF–Gel, (c) NP/Gel-1, (d) NP/Gel-2.

**Figure 5.**
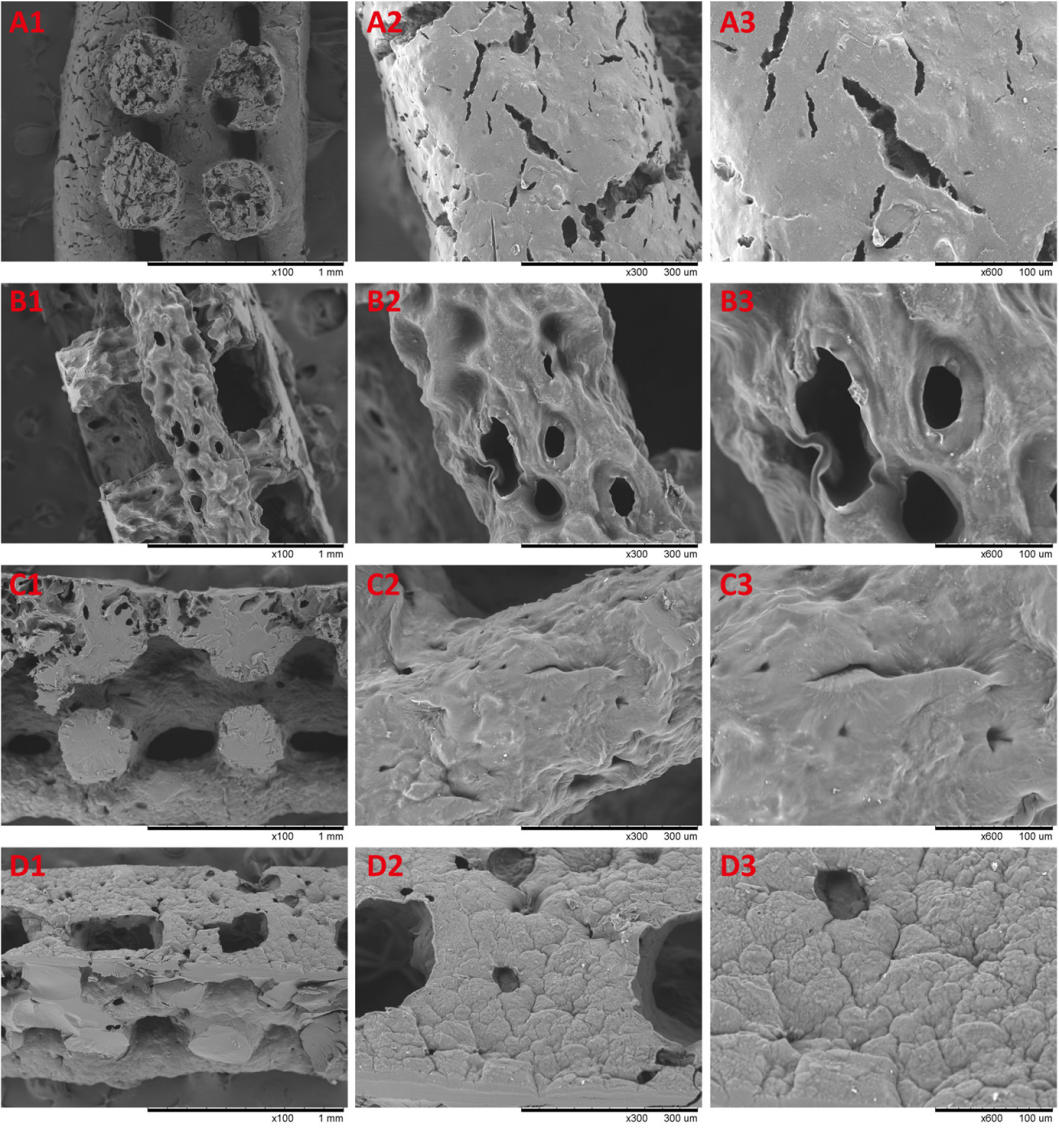
SEM images of 3D printed nanocomposite scaffolds. (a) NP/SF, (b) NP/SF–Gel, (c) NP/Gel-1, (d) NP/Gel-2.

Multiple layers of the polymer solution were deposited resulting in macroporous constructs. Pore geometry and overall strand deposition architecture were controlled by the parameter set of processing conditions. In this study, the inner diameter of the stainless steel needle was 520 *μ*m and the distance between two adjacent parallel strands was set at 1 mm. The printed deposition was followed by glutaraldehyde crosslinking of the construct and lyophilized in liquid nitrogen. Figure 4 shows that the 3D scaffolds possessed good pore interconnectivity and a highly regular deposition of strands regarding top view. Then the samples were observed by using scanning electron microscope to confirm the microstructures. Figure 5 shows the SEM micrographs of freeze-dried 3D nanocomposite scaffolds. Due to pre-mixing of the gelatin nanoparticles into the host matrix, the nanocomposite mixture was printed as a complete part. It was shown that AM led to a uniformly distribution of the nanomaterial in the printing media without any large agglomerates of nanoparticles. NP/SF, NP/SF–Gel and NP/Gel-1 had the same ratio of nanoparticles to host matrix (3/2), the NP of which completely melted in the printing media. Compared NP/Gel-1 (figure 5(c3)) with NP/Gel-2 (figure 5(d3)), notable differences were observed in the dispersion of nanoparticles within gelatin after printing. With the increase of NP to Gel ratio (3/2 to 3/1), it was observed that some portion of the particles does not melt during the AM process, but remain in their original form in the final-printed scaffolds. Thus, an adjustment of gelatin nanoparticles to host matrix ratio will lead to different density up to 75% or more for nanoparticle loadings. It is promising that adding nanomaterials can improve specific properties, such as mechanical properties, drug delivery and release kinetics properties, and tissue regeneration conductivity.

### 3.5. Viability and long-term proliferation of SMSCs cocultured with 3D scaffolds

**Figure 6.**
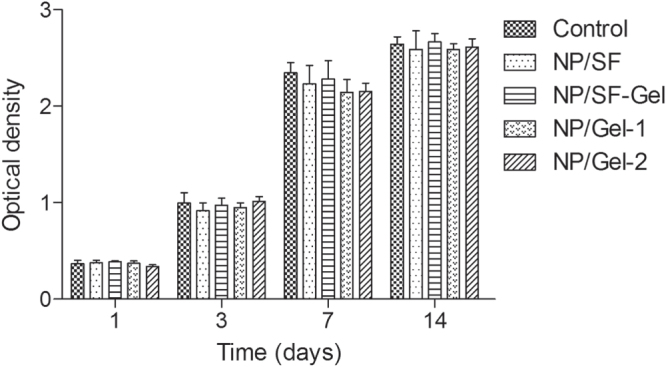
The proliferation of SMSCs cocultured with 3D printed nanocomposite scaffolds in the CCK-8 assay: the absorbance of these medium with CCK-8 was read at 450 nm.

**Figure 7.**
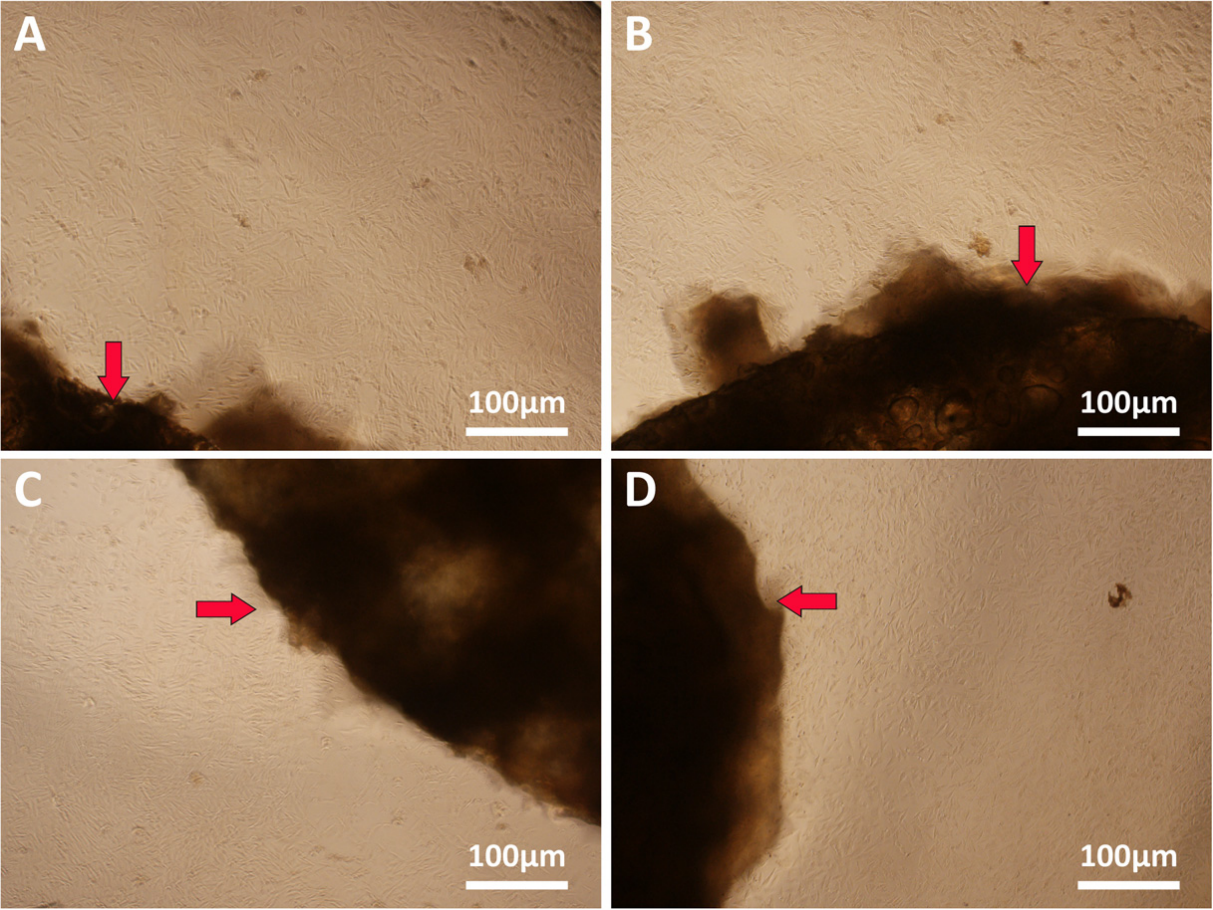
Microscopy images of SMSCs cocultured with 3D printed nanocomposite scaffolds shown by red arrows (a) NP/SF, (b) NP/SF–Gel, (c) NP/Gel-1, (d) NP/Gel-2.

The use of natural materials for preparing 3D scaffolds represents advantages in terms of biocompatibility. As shown in figure 6, there was no statistically significant difference between the 3D scaffolds group and the control group, indicating that 3D scaffolds have no toxicity on cell viability. Moreover, during the entire experimental period, SMSCs cultured with all samples displayed a significant increased viability profile. After 14 days *in vitro* culture, there was no statistically significant difference among NP/SF, NP/SF–Gel, NP/Gel-1 and NP/Gel-2 groups. We ascribe this to the excellent biocompatibility of gelatin and silk fibroin. We observed the SMSCs’ behavior under phase contrast microscope (figure 7). The cells in the four groups reached greater than 95% confluence and had an organized arrangement. All of them were plastic adherent with an elongated or spindle-shaped morphology, which is typical for SMSCs in appearance. Moreover no hypertrophic differentiation was observed in any of the groups, indicating that the cell–biomaterial interactions have no negative effect on cell behavior. The SMSCs cocultured with 3D printed scaffolds remained viable and proliferative.

## 4. Discussion

Stem cells, with their advantages of self-renewal and multi-potency, are thought to hold great promise in tissue engineering. Synovium-derived mesenchymal stem cells have emerged as a new cell source for musculoskeletal regeneration [20]. In this study, SMSCs were isolated from synovial membrane tissue and exhibited a high expandability in the culture flasks. A minimal amount of synovial membrane tissue yields an adequate number of cells. In the subpopulation culture, the SMSCs adhered to plastic and had an elongated or spindle-shaped appearance. In the surface epitope expression SMSCs have no distinction from other MSCs. To date no specific surface or cellular marker has been described to positively identify SMSCs. After induction in lineage-specific culture medium, SMSCs are capable of differentiating into several mesenchymal lineages, such as chondrocytes, osteocytes and adipocytes. Due to their ease of accessibility and the fact that they exhibit excellent pluripotency potential, SMSCs are ideal seed cells for tissue engineering.

Besides stem cells, scaffolds are an indispensable part of tissue engineering. Scaffolds are 3D biocompatible structures which can mimic the ECM properties, provide a template for cell attachment and stimulate tissue formation *in vivo*. Nanocomposite scaffolds have received significant attention because of their potential combination of properties from both the nanomaterials and the host materials matrix. In this study the integration of nanotechnology with AM has created wholly new nanocomposite scaffolds, adding gelatin nanoparticles to the printing host matrix. Our method provides a strategy for the design and fabrication of various 3D printed nanocomposite scaffolds. At the early stage, tissue regeneration happens at the periphery of scaffolds with a decreased ingrowth toward the inner parts. For continuous ingrowth of tissue, interconnected porosity is important. Open and interconnected pores allow nutrients and molecules to transport to inner parts of a scaffold to facilitate tissue ingrowth, vascularization, as well as waste material removal [21]. Therefore, through adding each new layer on top of the prior layer in the 3D printing process, macroporous scaffolds can be constructed. Various pore geometry and strand deposition architecture can also be realized by the parameter set of processing conditions to meet requirements for scaffolds in tissue engineering.

In the fabrication of nanomaterials, gelatin nanoparticles were prepared using a two-step desolvation method. Gelatin is a hydrolyzed protein derived from collagen, which is the major component of natural ECM. Compared with collagen, gelatin is economical and low-antigenic, and it has been manipulated to various forms depending on the process and widely employed in biological applications [22]. Nanoparticles have been intensively used for applications in sensing, nanoelectronics, catalysis, biological imaging and drug delivery [23, 24]. Thus, the addition of prepared gelatin nanoparticles to AM printing media could enable the creation of entirely new nanocomposites possessing unique properties and lead to expansion of AM application areas. This union of nanotechnology and AM could offer clear advantages for the manipulation of fundamental material properties in 3D printed nanocomposite scaffolds by varying the ratio of nanoparticles to the host matrix, which can possess customized properties.

In the previous study [25], gelatin was blended with other organic or inorganic biomaterials to fabricate 3D scaffolds by various approaches for skin, cartilage and bone tissue engineering applications. Silk fibroin (SF) as a natural protein has also been widely used in tissue engineering due to its several unique properties including good biocompatibility, good oxygen and water vapor permeability, biodegradability, and commercial availability at a relatively low cost [26–30]. In this study silk fibroin (SF), gelatin (Gel), or a SF–Gel mixture, as the host materials matrix, exhibited their printability and solubility in water, as well as good combination with gelatin nanoparticles. The 3D printed nanocomposite scaffolds possessed a good biocompatibility in the process of coculturing with SMSCs and had no negative effect on cell behavior. The combination of SMSCs and nanocomposite scaffolds can be used for practical applications in tissue engineering in the future.

## 5. Conclusions

In the present study, we isolated synovium-derived mesenchymal stem cells (SMSCs) from synovial membrane tissue and characterized stem-cell properties. SMSCs are similar to other MSCs in their surface epitope expression, which are negative for CD45 and positive for CD44, CD90, and CD105. After incubation in lineage-specific medium, SMSCs could differentiate into chondrocytes, osteocytes and adipocytes. Gelatin nanoparticles (NP) were prepared using a two-step desolvation method and then pre-mixed into different host matrix to print various 3D printed nanocomposite scaffolds, which are biocompatible with SMSCs. In the future, the combination of SMSCs and nanocomposite scaffolds can be used for practical applications in tissue engineering.
